# Alternative splicing of α1 and β1 sGC genes is altered in aortic disease

**DOI:** 10.1186/2050-6511-14-S1-P42

**Published:** 2013-08-29

**Authors:** Emil Martin, Eva Golunski, Iraida Sharina

**Affiliations:** 1 University of Texas Houston Medical School, Department of Internal Medicine, Division of Cardiology, Houston, Texas, USA

## Background

Soluble guanylyl cyclase (sGC) plays an important role in cardiovascular homeostasis and its activity is crucial for maintaining vascular plasticity. Diminished sGC expression and activity is associated with the development of endothelial dysfunction and increase in vascular stiffness. We have previously demonstrated that alternative splicing of sGC transcripts produces sGC splice forms with altered enzymatic and regulatory properties. However, splicing of sGC genes in human aortic tissue was never investigated.

## Results

We demonstrate that α1 and β1 sGC genes undergo alternative splicing in human aorta. In normal aortas we detected expression of four different transcripts for α1 and β1 sGC genes, each encoding an alternative protein isoform. Next, we investigated if sGC splicing is altered in vascular disease. Total RNA was isolated from samples collected from patients who underwent aortic repair surgery due to damages associated with aortic diseases (aortic dissection, aortic aneurism or advanced atherosclerosis). Age and gender-matched aortic controls from cardiovascular (CV) healthy donors obtained from commercial sources (Capital Biosciences, MD) were used for comparison. Semi-quantitative RT-PCR analysis with primers specific to individual sGC transcripts demonstrated increased expression of inhibitory α1 sGC splice variants (α1-Transcript 6 and α1-Transcript 7, NCBI) in samples obtained from aortas of patients with descending and ascending aortic aneurisms. N1-α1 splice isoform, encoded by α1-Tr6, was previously demonstrated to have a dominant-negative phenotype, when co-expressed with canonical α1 and β1 subunits. In presented study, we characterized the activity of sGC heterodimer containing α1-isoform D (α1-Tr7) in Cos-7 cells. Although α1-isoform D formed a heterodimer with β1 sGC, the resulting enzyme had very low activity and significantly reduced response to major sGC modulators, including NO-donors, BAY41-2272, HMR1766 and cobinamide. Thus, increased expression of α1-Tr7 D should decrease sGC function in human aorta. On the another hand, expression of oxidation resistant isoform C-α1 sGC, encoded by α1-Tr5, was reduced in diseased samples as compared with healthy ones. The difference was most pronounced between descending aortic aneurism samples and normal controls. Western blot analysis of aortic lysates showed decreased level of 50 kDa C-α1 protein, confirming that C-α1 expression is impaired in diseased samples. We previously demonstrated that oxidative stress induces the expression of C-a1 sGC in human cancer cells through modulation of splicing. Here, we investigated if similar process occurs in vascular smooth muscle cells (VSMC) treated with hydrogen peroxide. Indeed, human aortic VSMC from cardiovascular healthy donor showed induction of C-α1 transcript in response to the treatment. This result allows us to suggest that preferential splicing of C-α1 supports sGC activity in oxidative environment and its deregulation contributes to vascular disease. In addition, q-PCR analysis with commercial assays targeted to the conserved transcripts sequences demonstrated increase in total α1 and β1 sGC mRNA. Thus, our data indicate that not changes in transcription, but rather deregulation of splicing of sGC genes, might be a contributing factor to diminished sGC function in diseased aortas.

## Conclusion

Our data indicate that alternative splicing is one of the mechanisms regulating sGC function in human aorta and could serve as potential target for new therapeutics.

